# Synthesis, Crystal Structure, and Electrochemical Investigation of a New Trithiane-Derived Compound for High-Performance Supercapacitors

**DOI:** 10.1021/acsomega.5c05184

**Published:** 2025-09-03

**Authors:** Afike Ayça Özen, Tolga Göktürk, Tuncer Hökelek, Ramazan Güp, Cansu Topkaya, Ayşe Gül Bilge, Sema Aslan

**Affiliations:** † Department of Chemistry, 229199 Mugla Sitki Kocman University, Mugla 48000, Türkiye; ‡ Department of Physics, Hacettepe University, Beytepe-Ankara 06800, Türkiye

## Abstract

A novel trithiane-based compound, 6,6′,6″-(1,3,5-trithiane-2,4,6-triyl)­tris­(2-methoxyphenol) (TTMP), was synthesized and comprehensively characterized to explore its potential as a high-performance supercapacitor electrode material. The molecular structure was elucidated by FT-IR, ^1^H and ^13^C NMR, MALDI-TOF-MS, and single-crystal X-ray diffraction, revealing a tetragonal crystal system with space group *I–4*. Hirshfeld surface analysis highlighted dominant H···H (43.8%), H···C/C···H (24.1%), H···O/O···H (16.4%), and H···S/S···H (13.4%) interactions, confirming that van der Waals forces and hydrogen bonding govern the crystal packing. Interaction energy calculations demonstrated that dispersion forces primarily contribute to the lattice stabilization. Electrochemical evaluation of TTMP-modified glassy carbon paste electrodes (TTMP/GCPE) indicated efficient charge transfer kinetics with a significant reduction in charge transfer resistance (1.57 vs 5.93 kΩ for bare GCPE). Supercapacitor performance tests conducted in 3 M KOH revealed a high specific capacitance of 2807.74 F/g at 5 mV/s and outstanding long-term cycling stability, with a capacitance retention exceeding 140% after 5000 cycles. The results suggest that TTMP exhibits synergistic electric double-layer capacitance and pseudocapacitive behavior, making it a promising candidate for next-generation energy storage systems.

## Graphical abstract

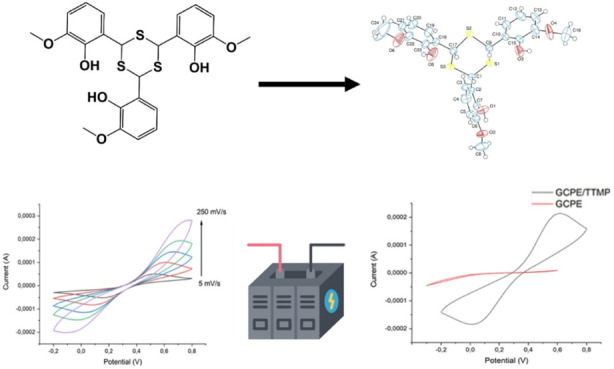

## 1 Introduction

Trithiane derivatives have gained increasing attention in materials science and electrochemistry due to their unique electronic properties and structural stability.^1^ The trithiane core, a six-membered saturated ring composed of alternating carbon and sulfur atoms, exhibits remarkable thermal and chemical stability, making it an ideal scaffold for molecular design.^2^ The presence of sulfur atoms in the trithiane framework provides a high electron density, enabling efficient charge transport and redox activity, both of which are critical for applications in energy-related systems.^3^ By introducing functional groups containing additional donor atoms such as oxygen, nitrogen, or sulfur, the electronic properties of trithiane derivatives can be finely tuned, enhancing their electrochemical performances and making them highly promising for next-generation supercapacitors.^4^

Supercapacitors, also known as electrochemical capacitors, have emerged as high-performance energy storage devices capable of delivering rapid charge/discharge rates, high power density and excellent cycling stability compared to conventional batteries.^5^ Unlike batteries, which rely on chemical reactions for energy storage, supercapacitors store energy through electrostatic charge accumulation (electric double-layer capacitance) or reversible redox reactions (pseudocapacitance).^6^ The choice of electrode materials plays a crucial role in determining the overall efficiency and energy density of the supercapacitors. Sulfur-containing materials, including trithiane-based compounds, have attracted significant interest due to their abilities to facilitate efficient charge transfer, stabilize electrode structures, and contribute to high capacitance through reversible redox reactions.^7^ One of the key challenges in supercapacitor technology is achieving an optimal balance between energy density, power density and long-term cycling stability. While carbon-based materials such as activated carbon and graphene are widely used in commercial supercapacitors due to their high surface areas and excellent conductivities, they often suffer from limited energy storage capacity.^8^ The incorporation of redox-active materials, including sulfur-rich organic compounds such as trithiane derivatives, can significantly enhance the charge storage mechanism by introducing additional faradaic contributions. Functionalized trithiane compounds, with their tunable donor properties and electron-rich nature, provide an excellent platform for designing novel electrode materials that combine the advantages of both electric double-layer capacitance and pseudocapacitance, leading to improved overall performance.^9^

Based on the available literature, researchers have investigated trithiane derivatives for supercapacitor applications. Notably, Pandey et al.^3^ reported the synthesis of a two-dimensional layered semiconducting coordination polymer (CuI-CP) constructed from 1,3,5-trithiane and copper iodide, which was systematically evaluated for its supercapacitor performance. Their study encompassed electrochemical techniques such as cyclic voltammetry, galvanostatic charge–discharge, and electrochemical impedance spectroscopy and also included the fabrication of a symmetric solid-state supercapacitor device utilizing a PVA-KOH gel electrolyte. Although other studies have explored trithiane derivatives in diverse electrochemical contexts-such as the electrochemical detection of nucleobases like adenine and guanine,^10^ or metal ion sensing including cerium,^11^ these works have largely remained peripheral to energy storage technologies. The investigation by Pandey et al.^3^ thus represents a pioneering step toward the application of trithiane-based systems as active components in supercapacitor devices.

Building upon these preliminary findings, the present work introduces a newly designed trithiane derivative featuring electron-donating methoxyphenol groups with the aim of enhancing redox activity and structural stability for energy storage applications. While prior research has primarily focused on trithiane-based coordination polymers or hybrid materials, there remains a critical lack of investigations into metal-free, structurally optimized trithiane compounds as stand-alone electrode materials. This study specifically addresses the research question: *Can a rationally designed, redox-active trithiane-based organic compound exhibit high capacitive performance and long-term cycling stability without metal incorporation?* To this end, the compound was synthesized through selective functionalization of the trithiane core and subjected to comprehensive structural and electrochemical analyses. These included crystal structure elucidation, intermolecular interaction mapping and electrochemical performance evaluation. The findings not only expand the scope of trithiane-based systems but also provide valuable insights into the relationship between the molecular structure and electrochemical behavior in sulfur-rich organic supercapacitor materials.

## 2 Experimental Section

### 2.1 Materials and Measurements

The Supporting Information provides concise descriptions and additional details regarding the materials, instruments used, experimental techniques employed for X-ray crystallography, electrochemical measurements and interaction energy calculations and energy frameworks.

### 2.2 Syntheses and Characterization

6,6′,6″-(1,3,5-Trithiane-2,4,6-triyl)­tris­(2-methoxyphenol) (TTMP) was synthesized as follows. 2-Hydroxy-3-methoxybenzaldehyde (152 mg, 1.0 mmol) and thioacetamide (75 mg, 1.0 mmol) were dissolved in 10 mL of ethanol (EtOH) under continuous stirring with a magnetic stirrer until a homogeneous solution was obtained. To this reaction mixture was added one drop of concentrated sulfuric acid (∼50 μL) and the reaction mixture was heated under reflux conditions with a condenser for 48 h. Upon completion of the reaction, the mixture was allowed to cool to room temperature, leading to the precipitation of the solid product. The precipitated product was filtered, washed with ethanol to remove impurities and then dried thoroughly to obtain TTMP (131 mg, 78%). X-ray quality crystals were obtained from ethanol:water (2:1) by slow evaporation at room temperature (Scheme 1).

**Scheme 1:**
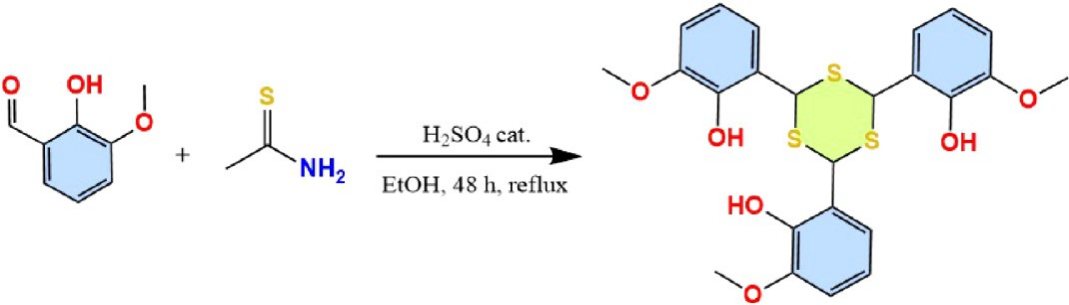
Synthesis of TTMP

### 2.3 6,6′,6″-(1,3,5-Trithiane-2,4,6-triyl)­tris­(2-methoxyphenol)

mp 290–292 °C; ^1^H NMR (400 MHz, DMSO-*d*_6_) δ = 9.24 (s, 3H; Ar–OH), 6.94 (m, 6H; Ar–H), 6.81 (t, 3H; Ar–H), 6.00 (s, 3H; CH), 3.80 ppm (s, 9H; CH_3_); ^13^C NMR (101 MHz, DMSO-*d*_6_) δ = 148.1, 143.1, 125.2, 120.9, 119.9, 112.2, 56.3 (CH_3_); 51.3 ppm; IR (ATR, cm^–1^): ν̃ = 3431 (O–H), 2934 (C–H), 1612 (C=C), 2369, 1210 (C–O); MALDI-TOF-MS: *m*/*z* calcd for [C_24_H_24_O_6_S_3_+Na]^+^: 527.063 [M + Na]^+^; found: 527.033; elemental analysis calcd (%) for C_24_H_24_O_6_S_3_: C 57.12, H 4.79, S 19.06, found: C 57.15, H 4.78, S 19.06.

## 3 Result and Discussion

### 3.1 Characterization of the Compound

The structural characterizations of TTMP were carried out using melting point determination, FTIR, NMR spectroscopy, mass spectrometry and elemental analysis besides the single-crystal X-ray diffraction techniques. The compound exhibited a sharp melting point at 290–292 °C, indicating a high purity. This was further supported by CHS elemental analysis, where the deviations between the calculated and found values were minimal: C (0.07%), H (0.63%) and S (0.84%). These results confirm the satisfactory elemental composition and purity of the synthesized compound.

In the FTIR spectrum of TTMP (Figure S1), a broad band at 3431 cm^–1^ was attributed to the presence of the phenolic groups. The absorption band at 2934 cm^–1^ is assigned to aliphatic C–H stretching of methoxy groups. A distinct band at 1612 cm^–1^ is attributed to aromatic C=C stretching, while the bands at 1269 and 1210 cm^–1^ are due to C–O stretching vibrations of the methoxy and phenolic groups, respectively.^12^

The ^1^H NMR spectrum of TTMP (Figure S2) shows a singlet at δ 9.24 ppm for the phenolic −OH protons (3H). Aromatic protons appear as two signals at δ 6.94 and 6.81 ppm, integrating for six protons. A singlet at δ 6.00 ppm is attributed to three protons on the sulfur-containing aromatic ring. The methoxy groups give a singlet at δ 3.80 ppm, corresponding to nine protons.^13,14^

The ^13^C NMR spectrum (Figure S3) of the TTMP displays aromatic carbon signals between 112.2 and 148.1 ppm, consistent with hydroxy- and methoxy-substituted benzene rings. The downfield signals at 148.1 (C–OH) and 143.1 ppm (C–OCH_3_) correspond to oxygen-substituted quaternary aromatic carbons. Peaks at 125.2, 120.9, and 119.9 ppm are assigned to aromatic CH carbons. The methoxy group appears at 56.3 ppm (O–CH_3_) and a nonprotonated tertiary carbon is observed at 51.3 ppm (quaternary), confirming the proposed structure.^15^

The calculated molecular mass of the TTMP was found to be *m*/*z* 527.063 [M + Na]^+^, while the experimentally observed mass in the MALDI-TOF-MS spectrum (Figure S4) appeared at *m*/*z* 527.033 [M + Na]^+^.

### 3.2 X-ray Structure

The X-ray structural determination of TTMP confirms the assignment of its structure from spectroscopic data. The asymmetric unit along with the atom-numbering scheme is depicted in Figure 1a. The experimental details and hydrogen bond geometry are given in Tables 1 and 2, respectively. The selected geometric parameters are given in Table S1.

**Table 1:** Experimental Details

Crystal data	
CCDC	2432665
Chemical formula	C_24_H_24_O_6_S_3_
*M*_r_	504.61
Crystal system, space group	Tetragonal, *I* −*4*
Temperature (K)	273 (2)
*a*, *c* (Å)	23.851 (3), 8.8343 (16)
*V* (Å^3^)	5025.7 (17)
*Z*	8
Radiation type	Mo *K*α
μ (mm^–1^)	0.33
Crystal size (mm)	0.11 × 0.09 × 0.08
Data collection	
Diffractometer	Bruker *APEX* II QUAZAR three-circle diffractometer
Absorption correction	–
No. of measured, independent and observed [*I* > 2σ(*I*)] reflections	28363, 5071, 3292
*R*_int_	0.078
(sin θ/λ)_max_ (Å^–1^)	0.624
Refinement	
*R*[*F* ^2^ > 2σ(*F* ^2^)], *wR*(*F* ^2^), *S*	0.052, 0.083, 1.02
No. of reflections	5071
No. of parameters	317
No. of restraints	3
H atom treatment	H atoms treated by a mixture of independent and constrained refinement
Δρ_max_, Δρ_min_ (e Å^–3^)	0.21, −0.21
Absolute structure parameter	0.48 (4)

**Table 2:** H-Bond Geometry (Å, °) for TTMP^a^,^b^

*D*—H···*A*	*D*—H	H···*A*	*D*···*A*	*D*—H···*A*
O1—H1*A*···O2	0.82	2.21	2.663 (4)	115
O1—H1*A*···S2^ii^	0.82	2.51	3.175 (3)	139
O3—H3*A*···O4	0.82	2.19	2.652 (4)	116
O3—H3*A*···O3^iii^	0.82	2.28	3.014 (4)	149
O5—H5*A*···S1^i^	0.90 (2)	2.79 (3)	3.465 (3)	134 (3)
O5—H5*A*···O6	0.90 (2)	2.12 (4)	2.636 (5)	116 (3)
C9—H9···O4^ii^	1.02 (3)	2.43 (3)	3.413 (5)	162 (2)
C13—H13···O5^iii^	0.93	2.57	3.386 (6)	146
C8—H8*A*···*Cg*4^iv^	0.96	2.95	3.761 (5)	143
C12—H12···*Cg*2^v^	0.93	2.92	3.807 (5)	159
C20—H20···*Cg*4^vi^	0.93	2.88	3.699 (4)	148

a Cg2 and Cg4 are the centroids of the C2···C7 and C18···C23 rings, respectively.

b Symmetry codes: (i) *x*, *y*, *z* + 1; (ii) −*y* + 1, *x*, −*z* + 1; (iii) *y*, −*x* + 1, −*z* + 1; (iv) −*y*, *x*, −*z* + 2; (v) −*x*, −*y*, *z* + 1; (vi) −*y* + 1/2, *x* – 1/2, −*z* + 5/2.

**Figure 1:**
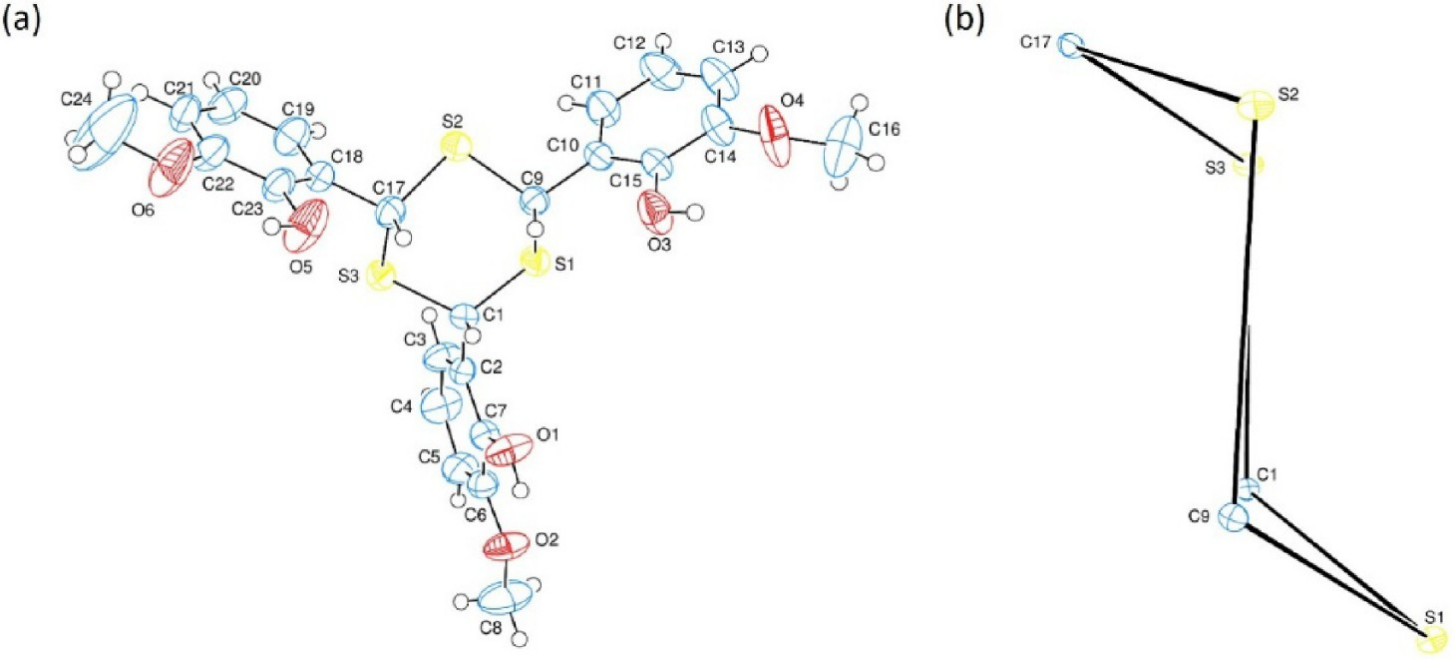
a The asymmetric unit of the TTMP with the atom numbering scheme. Thermal ellipsoids are drawn at the 50% probability level. b Conformation of the *D* (S1—S3/C1/C9/C17) ring.

The central six-membered trithiane, *A* (S1—S3/C1/C9/C17), ring is in chair conformation (Figure 1b) with the puckering parameters^16^ of *Q*_T_ = 0.8125(28) Å, θ = 176.49(20)°, and φ = 69(4)°. The S—C bond lengths and S—C—S and C—S—C bond angles vary between 1.811(5)—1.828(5) Å, 113.4(3)—115.5(2)°, and 99.7(2)—101.2(2)° with the average values of 1.821(5) Å, 114.2(2)°, and 100.5(2)°, respectively (Table S1). These average values are reported as 1.814(9) Å, 114.7(7)°, and 98.9(6)°, respectively, in 1,3,5-trithiane.^17^ A carbon valence angle exceeding the ideal tetrahedral angle is common in this class of cyclic compounds. For example, similar angular deviations have been reported in related structures: 114.5° in 1,3,5-trithioacetaldehyde,^18^ 112.5° in 1,4-dithiane,^19^ and 114.5° in the isostructural 1,3,5-triselenane.^20^

The planar six-membered, *B* (C2—C7), *C* (C10—C15), and *D* (C18—C23), rings arranged with dihedral angles of *B/C* = 68.32(12)°, *B/D* = 61.13(11)°, and *C/D* = 50.72(12)°. Atoms [C1, O1, and C8], [C9, O3, and O4], and [C17, O5, and C24] are [−0.0215(38), 0.0410(27), 0.0342(28), and 0.0095(50) Å], [0.0813(37), −0.0308(26), and −0.0452(31) Å], and [0.0491(37), −0.0249(37), 0.0363(35), and −0.0856(71) Å] away from the best least-squares planes of the rings *A*, *B*, and *C*, respectively. So, they are almost coplanar with the corresponding ring planes. The O—C bond lengths vary between 1.369(5)—1.435(6) Å with the average value of 1.380(6) Å. The O3—C15 [1.382(5) Å] and the O2—C8 [1.435(6) Å] bonds are the longest ones between the hydroxy and methoxy O—C bonds (Table S1). There are three intramolecular O–H···O hydrogen bonds (Table 2) between hydroxy and methoxy oxygens. In the crystal structure, intramolecular O–H···O and intermolecular O—H···S, O—H···O, and C–H···O hydrogen bonds connect molecules into a network facilitated by bifurcated hydrogen bonding interactions (Figure 2).

**Figure 2:**
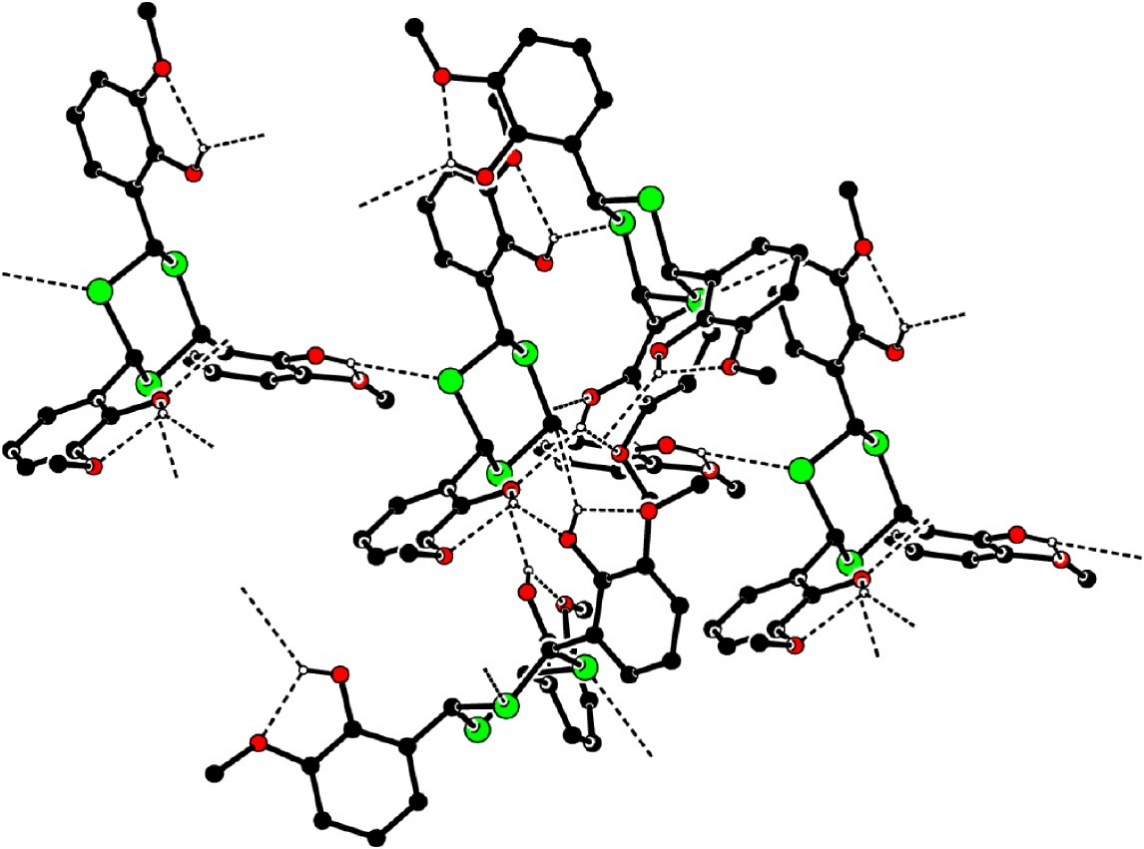
A partial packing diagram. Only the intramolecular O–H···O and intermolecular O–H···O and O–H···S hydrogen bonds are shown as dashed lines. The other H atoms have been omitted for the sake of clarity.

Furthermore, the three C—H···π interactions (Table 2) help the stabilization of the structure. The Flack absolute structure parameter^21^ was refined; expected values are 0 for the correct and +1 for the inverted absolute structure. The refined value is 0.48(4) (Table 1). Thus, the absolute structure is not determined reliably.

### 3.3 Hirshfeld Surface Analysis

To analyze intermolecular interactions in the TTMP crystal, a Hirshfeld surface (HS) analysis was performed using *CrystalExplorer 17.5.* ^22–24^ In the HS mapped over *d*_norm_ (Figure 3a), white areas represent contacts equal to the sum of van der Waals radii, while red and blue indicate shorter and longer contacts, respectively.^25^ Bright red spots correspond to the donor and acceptor regions. On the HS mapped over electrostatic potential (Figure 3b), these appear as red and blue regions, indicating negative and positive potentials.^26,27^

**Figure 3:**
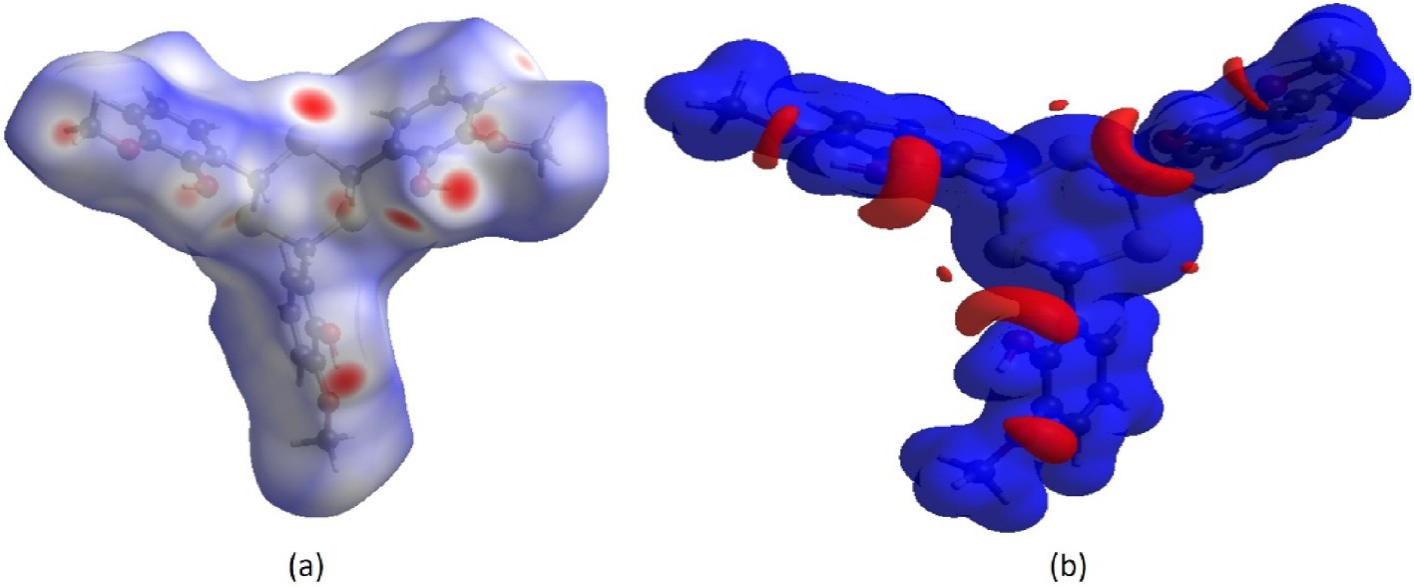
(a) Views of the three-dimensional Hirshfeld surfaces of TTMP plotted over *d*_norm_ in the ranges of −0.3564 to 1.8668 au. (b) A view of the three-dimensional Hirshfeld surface of the TTMP is plotted over electrostatic potential energy in the range of −0.0500 to 0.0500 au.

The blue and red regions on the electrostatic potential map represent positive (H-bond donors) and negative (H-bond acceptors) electrostatic potentials, respectively. π···π stacking and C–H···π interactions were further examined using a shape-index surface. This tool helps identify packing motifs, including planar stacking and aromatic interactions such as C–H···π and π···π. In the shape index, π···π stacking is indicated by adjacent red and blue triangles; the absence of such patterns suggests no π···π interactions, as confirmed in Figure 4a,b for TTMP. C–H···π interactions appear as “red π-holes,” reflecting interactions between CH groups and the centroids of nearby aromatic rings (Figure 4a,b).

**Figure 4:**
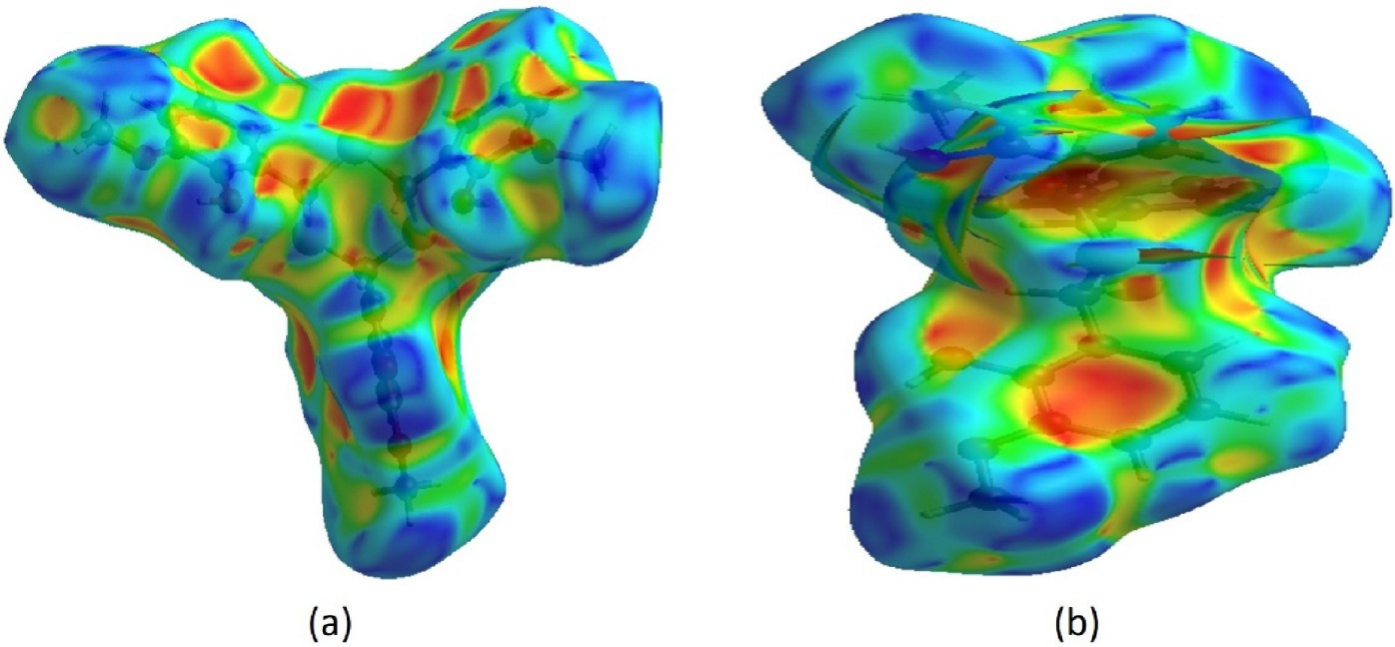
The Hirshfeld surface of the TTMP plotted over the shape index for two orientations, (a) front side and (b) back side.

The overall 2D fingerprint plot (Figure 5a) and along with the individual plots corresponding to H···H, H···C/C···H, H···O/O···H, H···S/S···H, C···O/O···C, O···O and O···S/S···O contacts^28^ are illustrated in Figure 5b–h respectively, along with their relative contributions to the Hirshfeld surface. Among these, H···H contacts are the most prominent, contributing 43.8% to the overall crystal packing (Table S1). This interaction is represented in Figure 5b by a broad distribution of high-density points, with a tip at *d*_e_ = *d*_i_ = 0.98 Å, which is attributed to the high proportion of hydrogen atoms in the molecule. H···C/C···H contacts, which are indicative of C—H···π interactions contribute 24.1% to the Hirshfeld surface and are characterized by a pair of distinct wings in the fingerprint plot, with tips located at *d*_e_ + *d*_i_ = 2.76 Å (Table S1 and Figure 5c). H···O/O···H interactions, contributing 16.4%, are represented by a pair of sharp spikes at *d*_e_ + *d*_i_ = 2.12 Å (Table S1 and Figure 5d). The fingerprint plot for H···S/S···H contacts (13.4% contribution, Table S1 and Figure 5e) displays a pair of longer spikes with tips at *d*_e_ + *d*_i_ = 2.37 Å. C···O/O···C contacts contribute 1.8% to the Hirshfeld surface (Figure 5f) and are visible as a pair of wings with tips at *d*_e_ + *d*_i_ = 3.38 Å. Lastly, the O···O (0.2%, Figure 5g) and O···S/S···O (0.2%, Figure 5h) interactions show very low point densities, indicating their minimal contribution to the overall packing.

**Figure 5:**
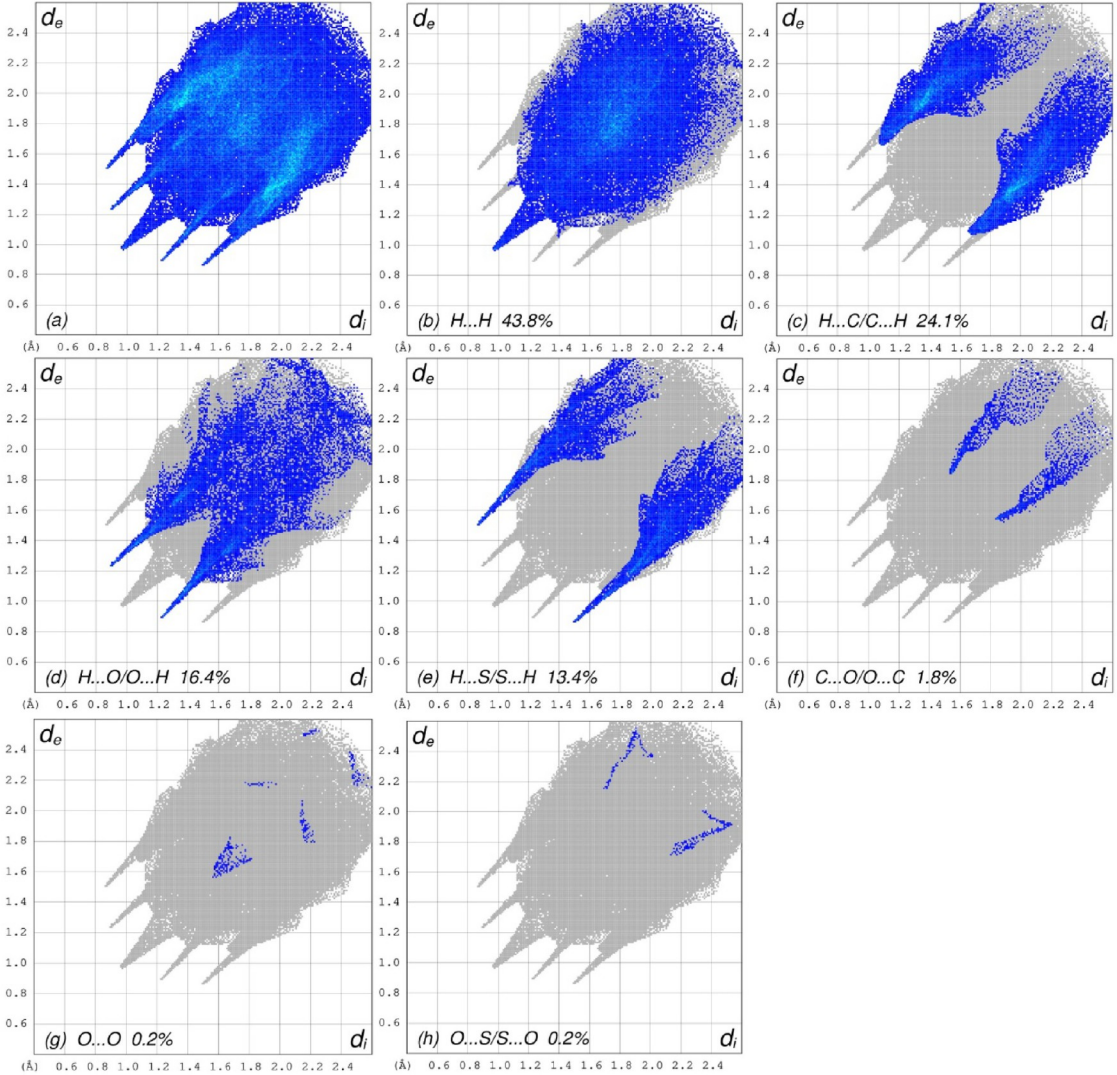
Full 2D fingerprint plots for TTMP showing (a) all interactions and delineated into (b) H···H, (c) H···C/C···H, (d) H···O/O···H, (e) H···S/S···H, (f) C···O/O···C, (g) O···O, and (h) O···S/S···O interactions.

The nearest-neighbor coordination environment of a molecule can be inferred from the color patches on the Hirshfeld surface (HS), which reflect the proximity of surrounding molecules. HS representations mapped with the *d*_norm_ function are presented for H···H, H···C/C···H and H···O/O···H interactions are shown in Figure 6a–d, respectively.

**Figure 6:**
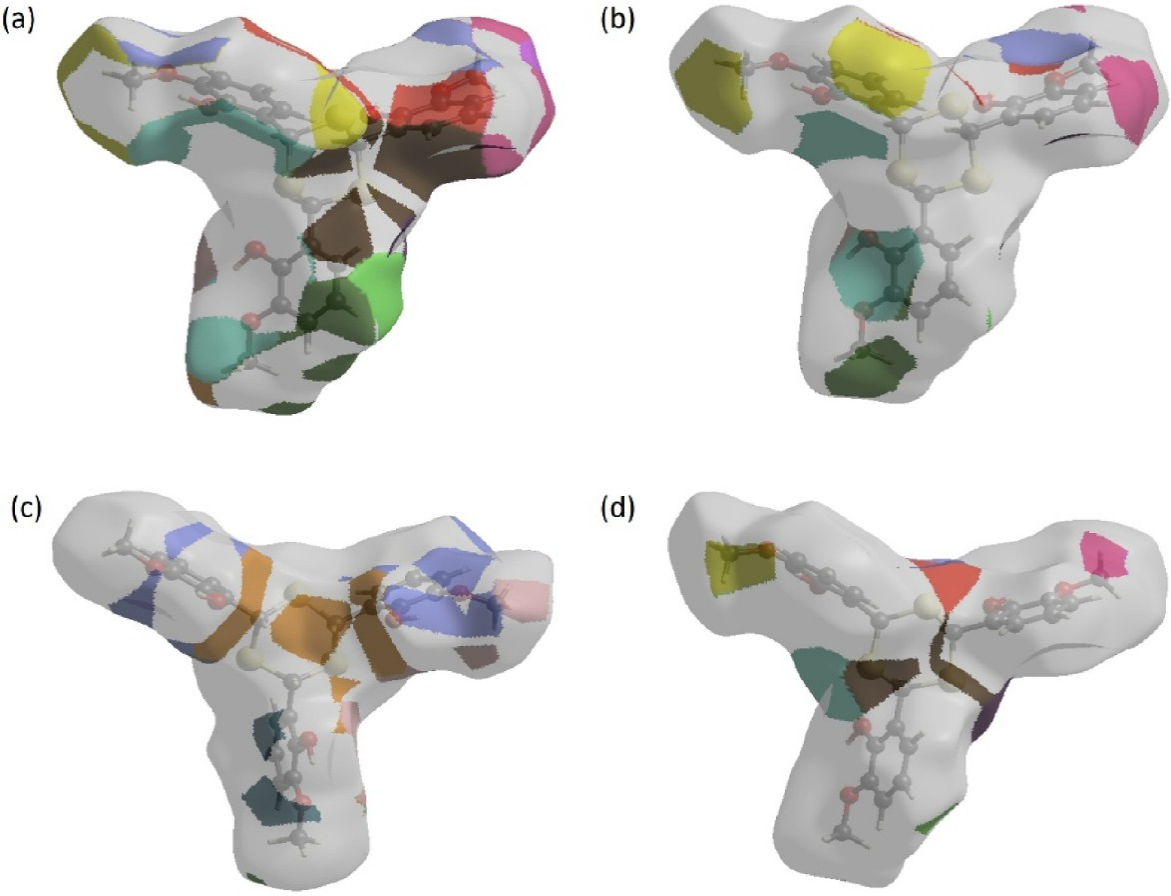
HS representations with *d*_norm_ plotted onto the surface for (a) H···H, (b) H···C/C···H, (c) H···O/O···H, and (d) H···S/S···H interactions.

The HS analysis confirms the critical role of hydrogen atom contacts in crystal packing. The prevalence of H···H, H···C/C···H, H···O/O···H, and H···S/S···H interactions indicates that van der Waals interactions and hydrogen bonding are primary contributors to the stabilization of the crystal structure.^29^

### 3.4 Interaction Energy Calculations and Energy Frameworks

The intermolecular interaction energies were calculated using the CE-HF/3-21G energy model implemented in *CrystalExplorer 17.5*.^24^ Hydrogen-bonding interaction energies (in kJ mol^–1^) were calculated to be [−48.6 (*E*_ele_), −2.5 (*E*_pol_), −61.2 (*E*_dis_), 60.0 (*E*_rep_), and −57.6 (*E*_tot_)] (for O3—H3A···O3), [−15.1 (*E*_ele_), −7.0 (*E*_pol_), −16.0 (*E*_dis_), 1.9 (*E*_rep_), and −32.7 (*E*_tot_)] (for C9—H9···O4), [−14.2 (*E*_ele_), −1.1 (*E*_pol_), −25.9 (*E*_dis_), 9.6 (*E*_rep_), and −30.8 (*E*_tot_)] (for O1—H1A···S2), [−6.6 (*E*_ele_), −8.3 (*E*_pol_), −18.2 (*E*_dis_), 6.5 (*E*_rep_), and −23.2 (*E*_tot_)] (for C13—H13···O5) and [−10.8 (*E*_ele_), −5.8 (*E*_pol_), −27.3 (*E*_dis_), 20.6 (*E*_rep_), and −22.7 (*E*_tot_)] (for O5—H5A···S1) hydrogen-bonding interactions.

Additionally, energy frameworks were visualized for electrostatic (red cylinders), dispersion (green cylinders), and total (blue cylinders) energies (Figure 7a–c). Analysis of these frameworks reveals that the dispersion energy contribution is the primary factor for stabilizing of the structure.

**Figure 7:**
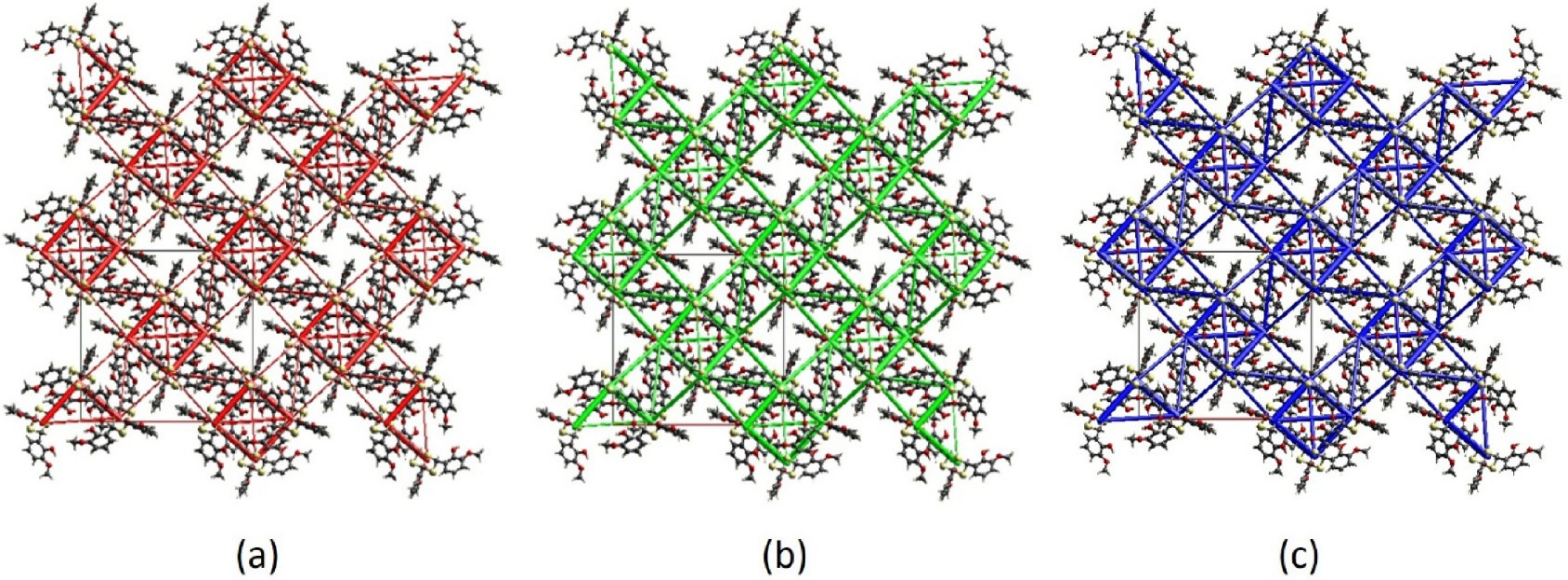
Energy frameworks for a molecular cluster of TTMP, viewed along the *c*-axis direction: (a) *E*_ele_, (b) *E*_dis_, and (c) *E*_tot_ diagrams.

### 3.5 Electrochemical Measurements

All electrochemical measurements were performed using a Gamry 1010E workstation, including cyclic voltammetry (CV), differential pulse voltammetry (DPV), galvanostatic charge/discharge (GCD), and electrochemical impedance spectroscopy (EIS). A three-electrode configuration was employed, consisting of a saturated calomel electrode as the reference, a platinum electrode as the counter, and a glassy carbon paste electrode (GCPE) modified with active material (TTMP) as the working electrode. The GCPE was prepared by mixing 80% GC powder with 20% mineral oil, while the optimal amount of TTMP was determined based on DPV measurements (Figure 8d).^30^

**Figure 8:**
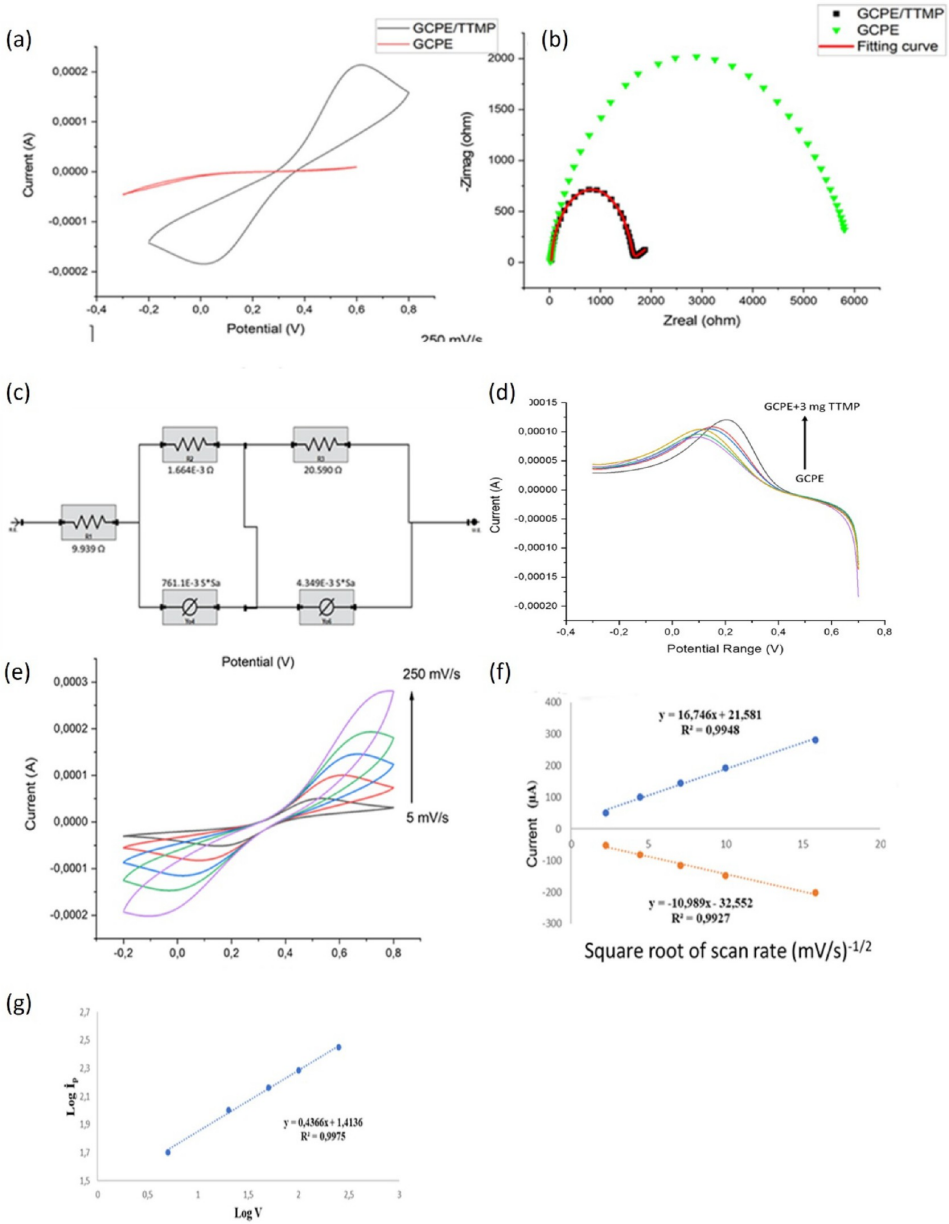
(a) CV and (b) EIS comparisons of TTMP-modified and plain GCPEs, including (c) a Nyquist plot fitting curve and circuit. (d) Differential pulse voltammograms for the determination of the optimum TTMP/GCPE ratio. (e) CV voltammograms of the TTMP/GCPE electrode at increasing scan rates and the charge transfer mechanism kinetics graphs for (f) square roots of the scan rates and (g) logarithms of the scan rates are given.

After the electrode surface was polished to achieve a smooth finish, the TTMP/GCPE electrode was employed as the working electrode in a standard three-electrode system. Different electrolytes were used for electrochemical characterization and supercapacitor performance testing. For characterization and optimization, a redox probe solution containing [Fe­(CN)_6_]^3–/4–^ (5.0 mM in 0.1 M KCl) was utilized to evaluate the electrode’s electron transfer kinetics. In contrast, supercapacitor measurements were performed in a 3.0 M aqueous KOH solution. Prior to testing, the working electrode was immersed in the electrolyte for 10 s to ensure adequate surface wetting and to allow equilibrium to be established. Electrochemical measurements were conducted within a potential window of −0.3 to +0.7 V, while cyclic voltammetry (CV) was recorded between −0.2 and +0.8 V. Electrochemical impedance spectroscopy (EIS) was carried out over a frequency range of 10^–2^ to 10^5^ Hz. GCD measurements were recorded under a 0.1 A/cm^2^ current density. Details of the structural representation methods and calculation formulas are provided in the Supporting Information.

#### 3.5.1 Electrochemical Characteristics of TTMP

CV and EIS methods are common methods to evaluate the charge transfer mechanisms of electrochemically active candidates. Therefore, the charge transfer mechanisms of the TTMP-modified GCPEs were investigated with respect to the surface electronic behaviors by these methods. Initially, CV and EIS measurements of the TTMP/GCPE were conducted, and the circuit of the electrode was obtained. According to the CV results, TTMP/GCPE showed higher *I*_pa_ and Ipc values compared to the plain GCPE electrode, indicating that TTMP is showing activeness. Meanwhile, the CV results were compatible with the Nyquist plots of the EIS results. Figure 8a,b shows the CV and EIS results with the fitting circuit model [R (RQ)­(RQ)] (Figure 8c). The EIS diagram can be evaluated upon changing charge transfer resistance values (*R*_ct_), which are higher at low charge transfer rates. So, one may expect that the higher CV current should meet with a lower *R*_ct_ value. Thus, while the *R*_ct_ value of the plain GCPE was 5.93 kΩ, TTMP/GCPE was 1.57 kΩ. Therefore, the charge transfer is facilitated by the addition of TTMP to the GCPE electrode. While 1.57 kΩ may seem high for inorganic systems, it represents a significant improvement over the bare electrode (5.93 kΩ) and is acceptable for organic electroactive materials. Herein the amount of TTMP was chosen as 3.00 mg (Figure 8d). TTMP-modified electrodes were tested by the DPV method. The optimum amount of the TTMP was decided by adding the increasing amounts as 1.0, 1.5, 2.0, 2.5 and 3.0 mg to the 80% part of the paste (as TTMP is in the amorphous phase), which was then filled into the electrode hole.^31^

Therefore, the anodic peak changes of the aforementioned amounts of TTMP to the GCPE structure were investigated. The interaction of the donor groups of the TTMP with the [Fe­(CN)_6_]^−3^/[Fe­(CN)_6_]^−4^ redox couple has enhanced the electron transfer capability. From 1.00 to 3.00 mg of TTMP, the anodic peak currents (*I*_pa_) were enhaced as 43.34, 43.83, 44.99, 48.25, 55.39 and 76.31 μA. The related DPV voltammograms and the optimization graph are given in Figure S5. The increasing amount of the TTMP resulted with the enhancement of the current value, so the electroactivity of the GCPE is elevated by the proposed material. Thus, 3.00 mg of TTMP was chosen as the optimum amount for further studies.

Further, the charge transfer mechanism of the TTMP was evaluated by applying increasing scan rates on the electrode with a CV. Figure 8e exhibits the 3.00 mg TTMP including GCPE electrode CV performances from 5 to 250 mV/s scan rates (from −0.3 to +0.7 V potential window with the step size of 10 mV per second). The supporting electrolyte was 0.10 M KCl and 5.00 mM [Fe­(CN)_6_]^−3^/[Fe­(CN)_6_]^−4^ including 50.00 mM PBS solution (pH 7.00). The CVs and the related graphs are given in Figure 8e–g. According to the scan rate (mV/s), *I*_pa_ (μA), *I*_pc_ (μA), *E*_pa_ (V), *E*_pc_ (V), and Δ*E*_p_ (V) values given in Table 3, further calculations were conducted to enlighten the charge transfer mechanism.

**Table 3:** Current and Potential Values Evaluation for the TTMP/GCPE Electrode

Scan rate (mV/s)	*I*_pa_ *I*(μA)	*I*_pc_ (μA)	*E*_pa_ (V)	*E*_pac_ (V)	Δ*E*_p_ (V)
5	50.48	–51.31	0.53	0.15	0.38
20	100.11	–82.56	0.61	0.08	0.53
50	145.80	–115.10	0.67	0.02	0.65
100	193.16	–147.05	0.72	–0.03	0.75
250	281.33	–201.80	0.79	–0.10	0.89

Since the donor-functionalized trithiane derivatives have been shown to enhance conductivity, facilitate charge storage, and improve the stability of electrode materials during prolonged cycling due to the strong electron-donating effects of sulfur atoms, they promote redox activity and efficient ion transport within the electrode structure. It was observed that the difference between current potential and peak potentials (Δ*E*_p_) (Table 3) increased linearly with the increase of scanning rate. When forward scans were examined, it was determined that there was a linear relationship between log *I*_pa_ (μA) and log υ (mV/s) (Figure 8g). The regression equations obtained for 3.00 mg of TTMP/GCPE from the graph plotted against the square root of the scan rates of the currents shown in Figure 8f are calculated as *y* = 16.746*x* + 21.581 (*R* ^2^ = 0.9948) for the anodic region and *y* = −10.989*x* – 32.552 (*R* ^2^ = 0.9927) for the cathodic region. Here *y* represents the peak current (*I*_pa_ or *I*_pc_ in μA), and *x* denotes the square root of the scan rate (*v* ^1/2^ in (mV/s)^1/2^). Using these slope values, the ratio of slopes (*m*_c_/*m*_a_) was calculated as 0.656, indicating that the system is a partially reversible process on the electrode surface.^32^ The further calculation in Figure 8g, the expession of the plot was “log *I*_pa_ = 0.4366 log υ + 1.4136” with *R* ^2^ = 0.9975. The slope value of 0.4366 was found to be close to the theoretically expected value of 0.5 for diffusion-controlled electrode processes. Therefore, it indicates that substance transfer proceeds with a diffusion-controlled mechanism.^31^

#### 3.5.2 Supercapacitor Characteristics of TTMP

In Figure 9, the capacitive properties of 3 mg TTMP/GCPE are evaluated by examining the CV curves and GCD obtained at different scanning rates in the potential range of −0.2 to +0.8 V. The CV curves (Figure 9a) exhibited quasi-rectangular shapes with redox peaks, indicating a combined electric double-layer capacitance (EDLC) and pseudocapacitive behavior due to the redox-active sulfur and methoxyphenol groups in TTMP.^33^ The analyses showed that the increase in the scanning rate (Figure 9a) caused a significant increase in current values. It was observed that the CV curve area values (*i* × *A*) obtained at different scanning rates were increased linearly with the increasing scan rate. These data were graphed by placing the *y*-curve area values and the square root of the scanning rate to the *x*- and a slope value of 0.0181 was obtained from the graph (Figure 9b). In addition, the general status of this correlation coefficient (*R* ^2^) was calculated as 0.9943. These high credit conditions show that the electrode works regularly and shows diffusion-controlled character. *C*_sp_ values were calculated in the F/g unit related to the obtained data and the results are given in Table 4. Also, the TTMP/GCPE electrode at different scan rates was revealed with reversely increasing *C*_sp_ values. Especially at 5 mV/s, the electrode achieved a specific capacitance of 2807.74 F/g (Table 4), attributed to complete electrolyte ion penetration into the porous structure.^34^ At higher scan rates (e.g., 250 mV/s), *C*_sp_ decreased to 700.67 F/g due to limited ion diffusion time (Figure 9b).^35^ GCD measurements (Figure 9e) confirmed the CV results, with *C*_sp_ values 10% higher than CV-derived values (e.g., 3088.51 F/g at 1 mA/cm^2^), highlighting GCD’s reliability for quantifying Faradaic contributions.^36^

**Table 4:** *C*_sp_ and *E* Values of the TTMP/GCPE Electrode at Increasing Scan Rates

Scan Rate (mV/s)	$\sqrt{v}$	CV (A.V)	*C*_sp_ (F/g)	*E* (Wh/kg)
5	2.24	0.28	2807.74	758.09
20	4.47	0.32	2530.30	125.79
50	7.07	0.38	2121.80	57.29
100	10.00	0.42	1277.20	34.49
250	15.81	0.53	700.67	18.92

**Figure 9:**
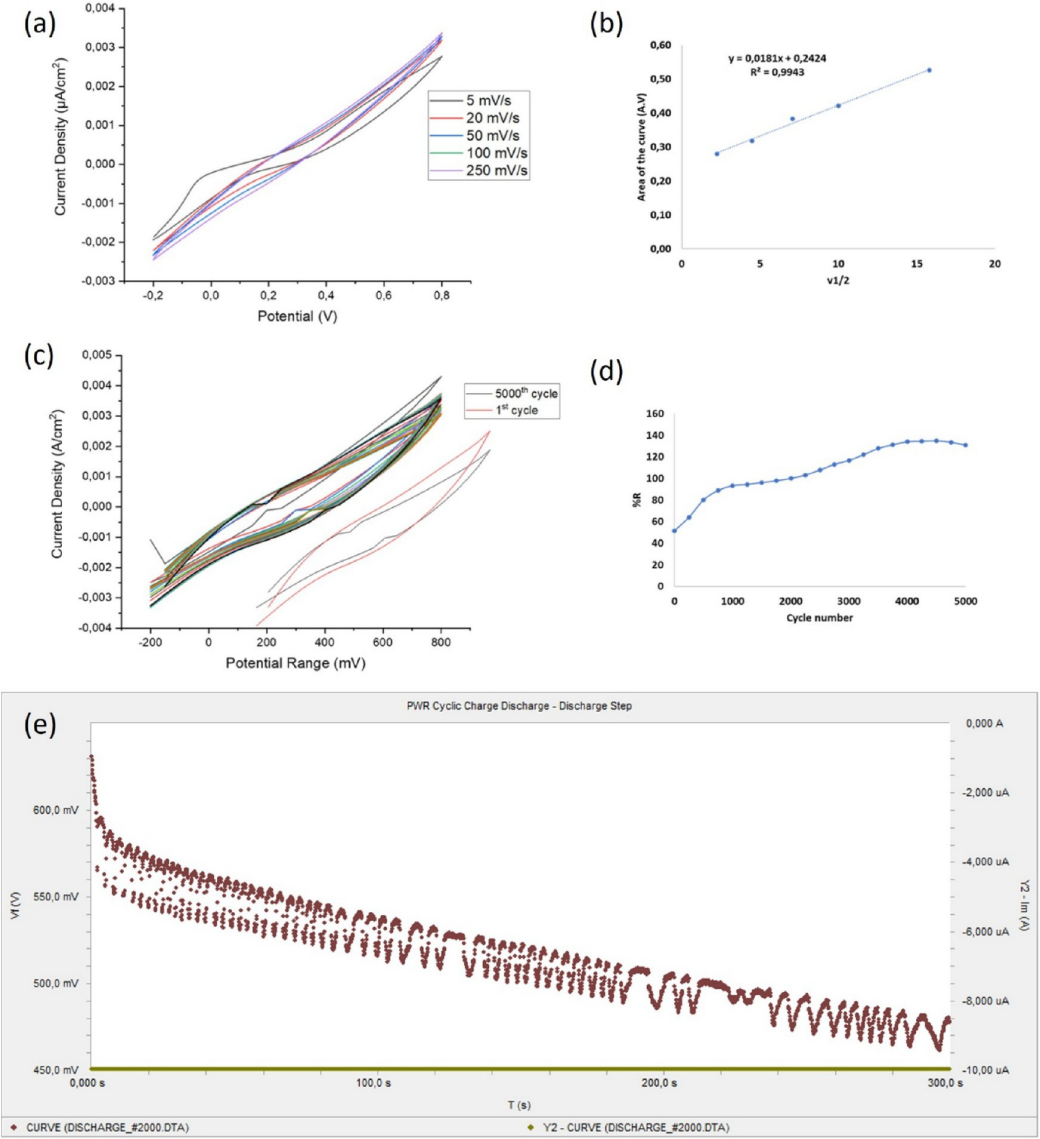
(a) CV measurements of the TTMP/GCPE electrode for increasing scan rates, (b) surface electron kinetics evaluation, (c) the long-term cycling performance for 5000 cycles with (d) *R*% *C*_sp_ values, and (e) GCD measurements.

This behavior is attributed to the fact that at lower scan rates, there is enough time for the deposition of the electrolyte ions to diffuse into the porous structure of the electrode material, allowing them to reach the inner surface area of the electrode. Also the calculations according to the Randles–Sewcik equation and the related graph showed a diffusion-controlled faradaic process. Therefore, these results pointed out that both EDLC and pseudocapacitance mechanisms can work effectively in this TTMP/GCPE composition. However, at the high scan rates, ions cannot penetrate deep into the pores due to time constraints, which limit the full use of the electrode surface. Only the outer surface or easily accessible regions contribute to charge storage.^37^

In order to evaluate the long-term performance of the energy storage capacity, a charge–discharge test of 5000 cycles was performed at a scanning rate of 100 mV/s. The voltammetric data recorded from the first to the 5000th cycle and the calculated *C*_sp_ values at every 250 cycles are presented comparatively in Figure 9c,d, respectively. Among the former cycles, *C*_sp_ values were measured at approximately 1500 F/g and increased with an increase in the number of cycles. From approximately the 2000th cycle onward, *C*_sp_ values started to show a stable trend approaching 4000 F/g. Later, the capacity stability was largely maintained until the 5000th cycle, and a slight decreasing trend was observed. These results show that the TTMP/GCPE electrode exhibits high stability in terms of long-term energy storage performance and the electrode surface adapts to the charge storage mechanism over time.

The recovery percentage (*R*%) plot for long-term charge–discharge cycles for 3 mg of TTMP/GCPE electrode showed a rapid increase from 60% at the beginning and reached 100% at the 1000th cycle (Figure 9d). After this point, the recovery rate gradually increased and stabilized at 140% around the 4000th cycle and this balance was maintained until the 5000th cycle. These results show that the electrode exhibits stable energy storage performance during long-term cycles, and the surface properties are preserved during the charge–discharge processes (Table 5). Over 5000 cycles, the capacitance retention exceeded 140% (Figure 9d), suggesting electrochemical activation of TTMP’s redox sites.^38^ Recovery efficiency reached 45% after resting, indicating reversible electrolyte rebalancing.^39^ In particular, the unusual increase in capacitance retention (up to 140%) during long-term cycling was attributed to progressive electrode activation and improved electrolyte diffusion, which were supported by kinetic analysis and CV behavior. The significance of achieving both high initial capacitance and long-term stability has been contextualized within current supercapacitor materials literature, reinforcing TTMP’s potential as a dual-behavior organic electrode material combining redox activity with electric double-layer capacitance.

**Table 5:** Comparative Summary of Sulfur- and Nitrogen-Containing Electrode Materials Used in Supercapacitor Applications

Electrode modification material	*C*_sp_ (F/g)	Type of the electrolyte	Cycle number	Reference
Schiff base polymers (DATP) and its metal complexes (M-DATPs, *M* = Cu^2+^, Fe^2+^)	853.87	6 M KOH	5000	^40^
Nickel and Nickel–cobalt [Ni_1/3_Co_2/3_(HL)_2_]·3H_2_O Schiff base complexes	683	6 M KOH	2000	^41^
Triazine-containing covalent organic polymers (TCOPs)	8412	KI-mixed H_2_SO_4_	3000	^42^
N/S codoped hollow porous carbon nanospheres	398	6 M KOH	10000	^43^
tetrachlorohydroquinone (TCHQ) and dichloroanthraquinone (DCAQ)/in metal-free medium	63	1 M H_2_SO_4_	>10000	^44^
poly(1,5-diaminoanthraquinone)	650	1 M H_2_SO_4_	25000	^45^
polyaniline–benzoquinone-hydroquinone composites	2646	1 M H_2_SO_4_	50000	^46^
TTMP/GCPE	2808	3 M KOH	5000	Present Study

A substantial body of experimental evidence demonstrates that small organic molecules can significantly enhance supercapacitor performance. For example, anthraquinone derivatives have been widely studied, with poly­(1,5-diaminoanthraquinone) delivering high conductivity and energy densities of approximately 46 Wh/kg,^44^ while systems pairing anthraquinone with 1,2-dihydroxybenzene have achieved specific capacitances of 63 F/g. Other redox-active organics, such as tetrachlorohydroquinone (TCHQ) and dichloroanthraquinone (DCAQ), have been employed in metal-free aqueous redox capacitors, demonstrating excellent cycling stability (>10,000 cycles) and energy densities near 14 Wh/kg.^44^ Similarly, functionalization of graphene-based materials with species like 2,5-dimethoxy-1,4-benzoquinone (DMQ) has yielded composite aerogels with specific capacitances up to 650 F/g and outstanding cycle life.^45^ Additional advances include the stabilization of polyaniline–benzoquinone–hydroquinone composites exhibiting specific capacitances of 2646 F/g at 0.5 mA/cm, and the use of supramolecular oligomers of 1,5-diaminoanthraquinone in 4 M H_2_SO_4_, achieving capacities in the range of 40–50 Ah/kg.^46^ Collectively, these studies clearly demonstrate the effectiveness of molecularly engineered redox-active organic systems in overcoming the inherent limitations of traditional electrode materials.

## 4 Conclusions

In this study, a novel trithiane-based compound, 6,6′,6″-(1,3,5-trithiane-2,4,6-triyl)­tris­(2-methoxyphenol) (TTMP), was successfully synthesized and structurally elucidated through spectroscopic and single-crystal X-ray diffraction techniques. The compound crystallizes in a tetragonal system of the *I–4* space group. Hirshfeld surface analysis revealed that hydrogen bonding and van der Waals interactions dominate the crystal packing. Energy framework calculations further emphasized the crucial role of the dispersion energy contribution in the stabilization of the structure.

Supercapacitor analyses performed at different scan rates showed that *C*_sp_ values for TTMP changed depending on the scan rates. At low scan rates, especially at 5 mV/s, a maximum Csp value of 2807.74 F/g was reached due to the better penetration of the electrolyte ions into the pores of the electrode material. This situation shows that both EDLC and pseudocapacitance mechanisms work effectively on the electrode surface. At higher scan rates, only the surface regions contributed to charge storage due to the limiting effect of ion diffusion kinetics, and this led to a decrease in *C*_sp_ values. Long-term charge–discharge performance showed a very interesting trend and the *C*_sp_ value, which was 60% at the beginning, increased by 140% between 4000th and 5000th cycles. The proposed supercapacitor showed a higher performance than those reported in the literature.^47–49^ This outstanding performance is attributed to the synergistic contribution of the electric double-layer capacitance and faradaic pseudocapacitance facilitated by the electron-rich sulfur-containing trithiane core and phenolic functionalities. The superior electrochemical performance is consistent with the Hirshfeld surface analysis, which revealed a crystal packing dominated by hydrogen bonding and van der Waals interactions, facilitating favorable ion-accessible pathways and efficient electrolyte penetration during charge–discharge processes. TTMP is found to be excellent in terms of electrochemical performance including higher specific capacitance, good rate performance, and cycle stability in 3 M KOH solution conditions. The integration of structural tunability and electrochemical responsiveness makes TTMP a strong candidate for next-generation energy storage applications. Future work will involve assembling symmetric and asymmetric supercapacitor devices using TTMP and testing them in real-world applications such as LED powering and extended cycle life up to 15000 cycles.

## Supplementary Material





## Data Availability

The data that support the findings of this study are available in the Supporting Information of this article.

## Notes

Materials, instruments used, experimental techniques employed for X-ray crystallography, electrochemical measurements, as well as the interaction energy calculations and energy frameworks. FTIR, ^1^H NMR, ^13^C NMR and MALDI-TOF-MS spectra of TTMP. Selected geometric parameters (Å, °) of TTMP (PDF)
(CIF)
