# Supplementary material for: Synthesis, Crystal Structure, and Electrochemical Investigation of a New Trithiane-Derived Compound for High-Performance Supercapacitors
Source: ACS Omega. 2025 Sep 3;10(36):41595–607. doi: 10.1021/acsomega.5c05184 (PMC12444602; doi:10.1021/acsomega.5c05184)
Supplement: Supplementary file 1 [file ao5c05184_si_001.pdf]

## Supplementary Information

# Synthesis, Crystal Structure, and Electrochemical Investigation of a New Trithiane-Derived Compound for High-Performance Supercapacitors

**Afike Ayça Özen[1], Tolga Göktürk[1*], Tuncer Hökelek[2], Ramazan Güp[1], Cansu Topkaya[1], Ayşe Gül Bilge[1], Sema Aslan[1*]**

*[1]Muğla Sıtkı Koçman University, Department of Chemistry, 48000 Muğla, Turkey*
*[2]Hacettepe University, Department of Physics, 06800 Beytepe-Ankara, Turkey*

**Corresponding author E-mail: tolgagokturk@mu.edu.tr, semaaslan@mu.edu.tr,*

## Table of Content

1. Materials and Methods……………………………………………………………..….2
2. X-ray Crystallography……………………………………………………………………2
3. Interaction Energy Calculations and Energy Frameworks………………………………….2
4. Electrochemical Measurements …………………………………………………………3
5. FTIR spectra of TTMP …………………………...……………………………………...4
6. $^{1}$H NMR spectra of TTMP ……………………………..……………………………..5
7. $^{13}$C NMR spectra of TTMP…………………………………….………………………...5
8. MALDI-TOF-MS spectra of TTMP………………………………………………………...6
9. Selected geometric parameters (Å, º) of TTMP. …….…………………………………...7
10. Differential pulse voltammograms for the determination of the optimum TTMP/GCPE ratio. …………………………... ……………………………………..................................7

## 1. Materials and Methods

All reagents were purchased from Merck/Aldrich and used without further purification. Microanalysis (C, N, H) were performed on a LECO 932 CHNS analyzer. $^{1}$H NMR spectra were recorded on a Bruker 400 MHz spectrometer in DMSO_$d_6$. IR spectrum was recorded on a pure solid sample with a Thermo-Scientific, Nicolet iS10-ATR from 4000 to 400 cm$^{-1}$ using ATR (attenuated total reflectance) technique. The melting point was obtained on an Electrothermal IA 9100 (UK) digital melting point apparatus and was uncorrected. Electrochemical studies were performed with a Metrohm Dropsense DRP-STAT-I400 Potentiostat/Galvanostat electrochemical workstation was equipped with a triple electrode system (Eco Chemie, Utrecht, The Netherlands) including glassy carbon paste electrode (GCPE) (20 mm diameter working electrode), Ag/AgCl (CH Instruments Inc. CHI111 reference electrode filled with 3 M KCl) and Pt wire counter electrode). Crystallographic data were recorded on a Bruker APEX-II CCD diffractometer using Mo Kα radiation (λ = 0.71073 Å).

## 2. X-ray Crystallography

The crystallographic data of compound were collected on a Bruker APEX II QUAZAR three-circle diffractometer using Mo Kα radiation (λ = 0.71073 Å) at 273 (2) K. The data were processed by SHELX program packages [SHELXT 2018/2[1] and SHELXL2018/3[2]] for solving and refining the structure, and ORTEP-3[3] and PLATON[4] programs were used in drawings. Atoms H1, H5A, H9 and H17 were located in a difference Fourier map, and refined isotropically. The other H atom positions were calculated geometrically at distances of 0.82 Å (for OH), 0.93 Å (for aromatic CH) and 0.96 Å (for CH3) and refined using a riding model by applying the constraints of Uiso(H) = k X Ueq (C, O), where k = 1.2 for aromatic CH and k = 1.5 for OH and CH3 hydrogens. Crystallographic data for the structure reported herein have been deposited with the Cambridge Crystallographic Data Centre as Supporting Information, CCDC No. **2432665**. Copies of the data can be obtained through application to CCDC, 12 Union Road, Cambridge CB2 1EZ, UK. (fax: +44 1223 336033 or e-mail: deposit@ccdc.cam.ac.uk or at http://www.ccdc.cam.ac.uk).

## 3. Interaction Energy Calculations and Energy Frameworks

The intermolecular interaction energies are calculated using CE-HF/3-21G energy model available in Crystal Explorer 17.5[5], where a cluster of molecules is generated by applying

crystallographic symmetry operations with respect to a selected central molecule within the radius of 3.8 Å by default.[6] The total intermolecular energy ($E_{tot}$) is the sum of electrostatic ($E_{ele}$),polarization ($E_{pol}$), dispersion ($E_{dis}$) and exchange-repulsion ($E_{rep}$) energies[7] with scale factors of 1.019, 0.651, 0.901 and 0.811, respectively.[8] Hydrogen-bonding interaction energies (in kJ $mol^{-1}$) were calculated to be [−48.6 ($E_{ele}$), −2.5 ($E_{pol}$), −61.2 (Edis), 60.0 ($E_{rep}$) and −57.6 ($E_{tot}$)] (for O3—H3A···O3), [−15.1 ($E_{ele}$), −7.0 ($E_{pol}$), −16.0 (Edis), 1.9 ($E_{rep}$) and −32.7 ($E_{tot}$)] (for C9—H9···O4), [−14.2 ($E_{ele}$), −1.1 ($E_{pol}$), −25.9 ($E_{dis}$), 9.6 ($E_{rep}$) and −30.8 ($E_{tot}$)] (for O1—H1A···S2), [−6.6 ($E_{ele}$), −8.3 ($E_{pol}$), −18.2 ($E_{dis}$), 6.5 ($E_{rep}$) and −23.2 ($E_{tot}$)] (for C13—H13···O5) and [−10.8 ($E_{ele}$), −5.8 ($E_{pol}$), −27.3 ($E_{dis}$), 20.6 ($E_{rep}$) and −22.7 ($E_{tot}$)] (for O5—H5A···S1) hydrogen-bonding interactions. Energy frameworks combine the calculation of intermolecular interaction energies with a graphical representation of their magnitude.[7] Energies between molecular pairs are represented as cylinders joining the centroids of pairs of molecules with the cylinder radius proportional to the relative strength of the corresponding interaction energy. Energy frameworks were constructed for $E_{ele}$ (red cylinders), $E_{dis}$ (green cylinders) and $E_{tot}$ (blue cylinders) (Figs. 8 a, b and c). The evaluation of the electrostatic, dispersion and total energy frameworks indicate that the stabilization is dominated via the dispersion energy contribution in the crystal structure of compound. The cylindrical radius is proportional to the relative strength of the corresponding energies and they were adjusted to the same scale factor of 80 with cut-off value of 5 kJ $mol^{-1}$ within 2 X 2 X 2 unit cells.

**4. Electrochemical Measurements**

A three-electrode system using Metrohm Dropsense DRP-STAT-I400 Potentiostat/Galvanostat was used for the electrochemical characterisations and supercapacitor measurements. Glassy carbon paste electrode (GCPE) was employed as working electrode, Ag/AgCl (3 M KCl filled) as reference and Pt wire was served as counter electrodes. GCPE was prepared by mixing 2 µm fine powder of glassy carbon particles with mineral oil by the ratio of 80/20 % (w/w) and filled into the electrode hole. Measurements were carried out in a 3 mL of 0.1 M KCl containing 50 mM phosphate buffer saline (PBS) solution. Also 5 mM $Fe^{2+/3+}$ redox probe solution was used for the electrochemical mechanism and electrochemical characterization studies. Supercapacitor measurements were carried out in 6 M KOH solution. Specific capacitance (Csp) and specific energy (E) calculations were processed for the supercapacitor studies according to the equations given in our previously reported study.[9]

## 5. FTIR spectra of TTMP

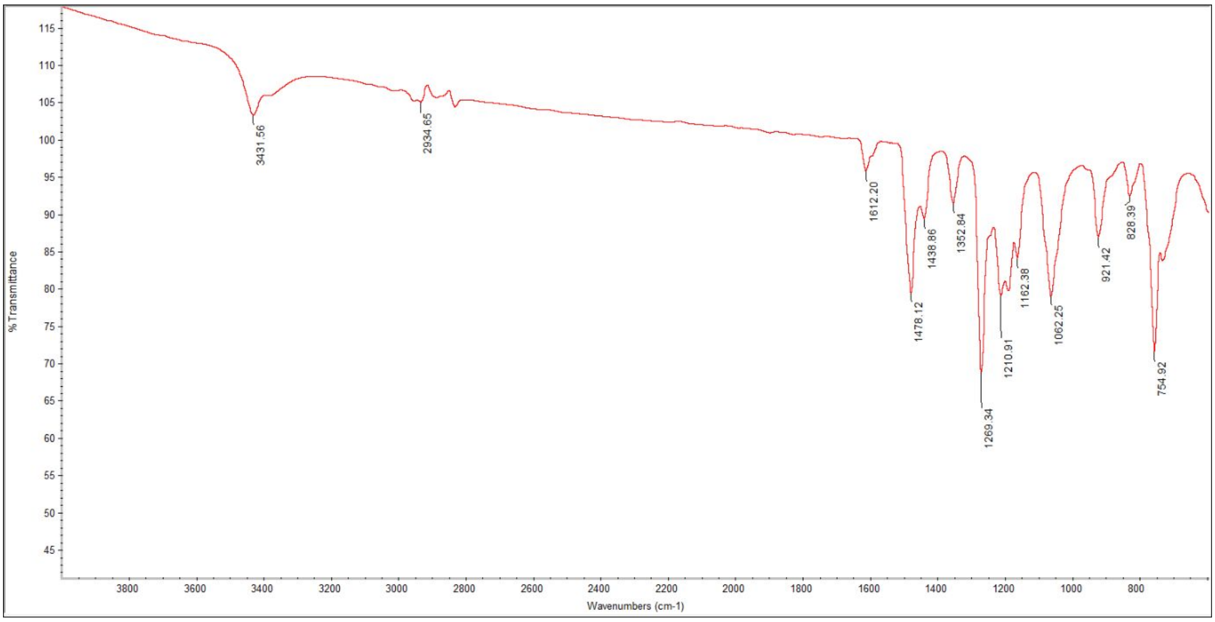


**Figure S1.** FTIR spectra of TTMP

## 6. $^1$H NMR spectra of TTMP

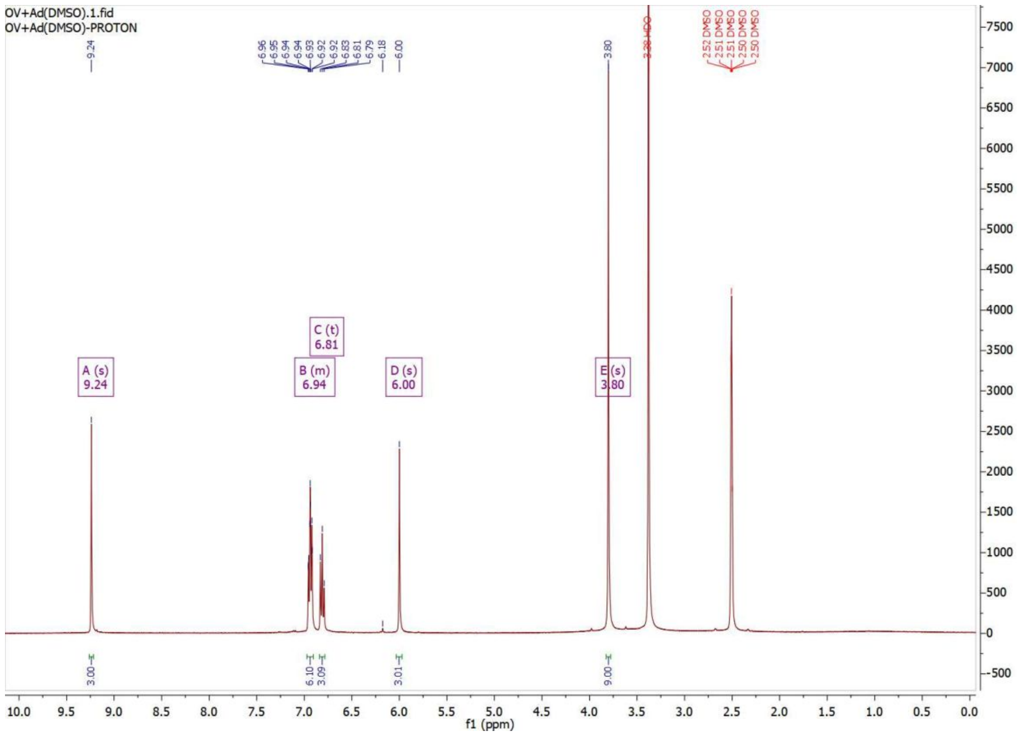


**Figure S2.** $^1$H NMR spectra of TTMP

## 7. $^{13}$C NMR spectra of TTMP

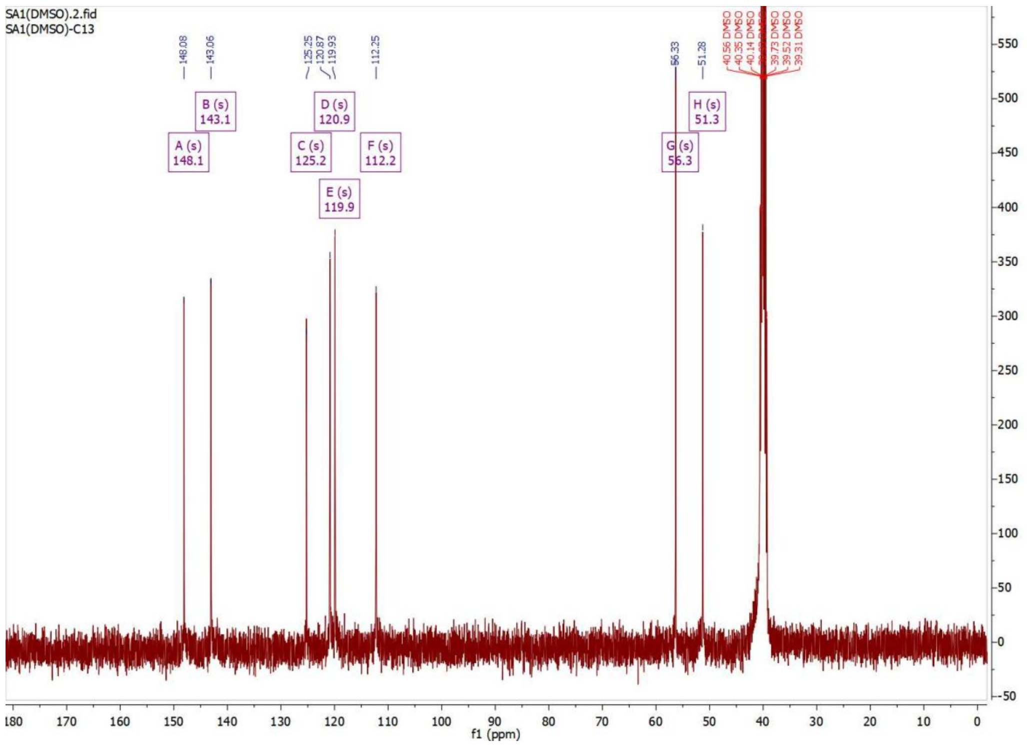


**Figure S3.** $^{13}$C NMR spectra of TTMP

## 8. MALDI-TOF-MS spectra of TTMP

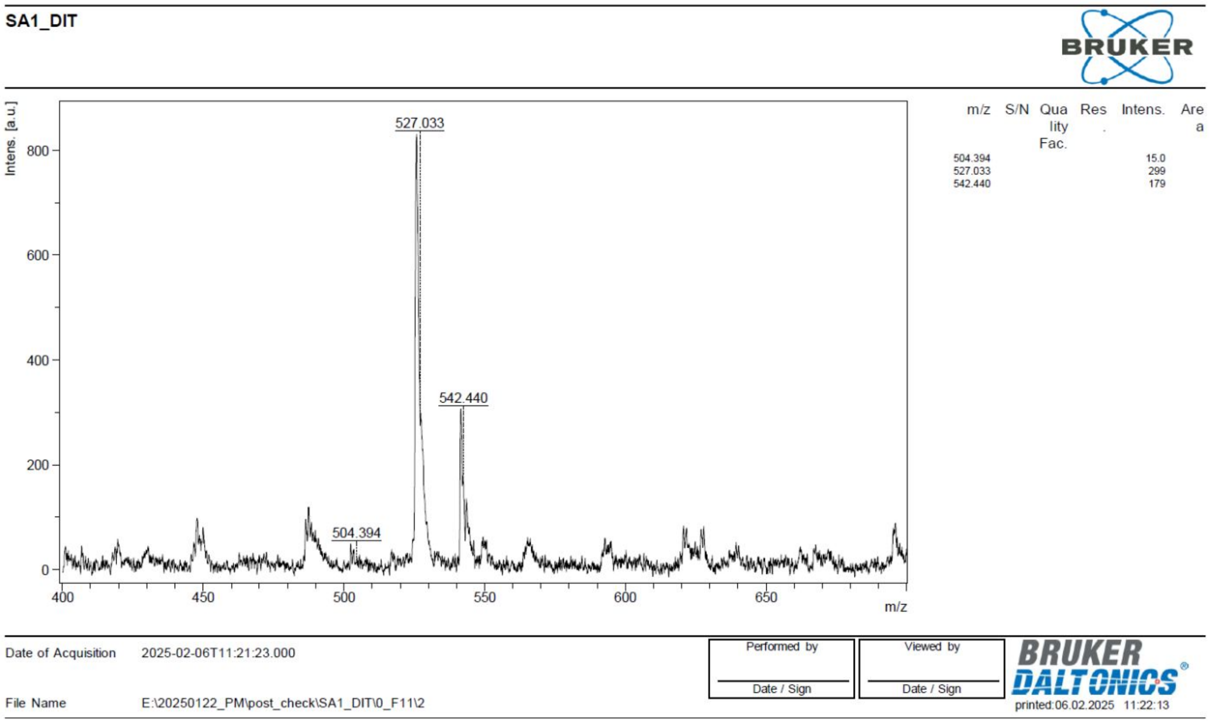


**Figure S4.** MALDI-TOF-MS spectra of TTMP

**Table S1.** Selected geometric parameters (Å, º).

| | | | |
|---|---|---|---|
| S1—C9 | 1.811 (5) | O2—C8 | 1.435 (6) |
| S1—C1 | 1.817 (5) | O3—C15 | 1.382 (5) |
| S2—C17 | 1.822 (5) | O4—C14 | 1.372 (5) |
| S2—C9 | 1.827 (5) | O4—C16 | 1.385 (7) |
| S3—C1 | 1.820 (5) | O5—C23 | 1.369 (6) |
| S3—C17 | 1.828 (5) | O6—C24 | 1.369 (8) |
| O1—C7 | 1.369 (5) | O6—C22 | 1.370 (6) |
| O2—C6 | 1.371 (5) | | |
| H5A···S1$^{i}$ | 2.79 (3) | O5···H17 | 2.39 (3) |
| S2···H11 | 2.91 | H13···O5$^{iii}$ | 2.57 |
| S2···H19 | 2.98 | O6···H5A | 2.12 (4) |
| H1A···S2$^{ii}$ | 2.51 | C5···H8C | 2.73 |
| S3···H3 | 2.91 | C5···H8B | 2.82 |
| O1···O2 | 2.663 (4) | C8···H5 | 2.54 |
| O3···O4 | 2.652 (4) | C13···H16B | 2.86 |
| O5···O6 | 2.636 (5) | C16···H13 | 2.83 |
| O1···H1 | 2.34 (3) | C21···H24B | 2.63 |
| O2···H1A | 2.21 | C24···H21 | 2.58 |
| H3A···O3$^{iii}$ | 2.28 | H5···H8B | 2.40 |
| O3···H9 | 2.41 (3) | H5···H8C | 2.28 |
| O4···H3A | 2.19 | H24A···H19$^{i}$ | 2.19 |
| H9···O4$^{ii}$ | 2.43 (3) | H21···H24B | 2.18 |
| C9—S1—C1 | 100.6 (2) | S1—C1—S3 | 115.5 (2) |
| C17—S2—C9 | 99.7 (2) | S1—C9—S2 | 113.7 (2) |
| C1—S3—C17 | 101.2 (2) | S2—C17—S3 | 113.4 (3) |

Symmetry codes: (i) $x$, $y$, $z$+1; (ii) −$y$+1, $x$, −$z$+1; (iii) $y$, −$x$+1, −$z$+1.

## References


1. Sheldrick, G. M. SHELXT – Integrated Space-Group and Crystal-Structure Determination. Acta Crystallogr. A 2015, 71, 3–8. DOI: 10.1107/S2053273314026370

2. Sheldrick, G. M. Crystal Structure Refinement with SHELXL. Acta Crystallogr. C 2015, 71, 3–8. DOI: 10.1107/S2053229614024218.

3. Farrugia, L. J. WinGX and ORTEP for Windows: An Update. J. Appl. Crystallogr. 2012, 45, 849–854. DOI: 10.1107/S0021889812029111.

4. Spek, A. L. Structure Validation in Chemical Crystallography. Acta Crystallogr. D 2009, 65, 148–155. DOI: 10.1107/S090744490804362X.

5. Turner, M. J.; McKinnon, J. J.; Wolff, S. K.; Grimwood, D. J.; Spackman, P. R.; Jayatilaka, D.; Spackman, M. A. CrystalExplorer17; The University of Western Australia: Australia, 2017.

6. Turner, M. J.; Grabowsky, S.; Jayatilaka, D.; Spackman, M. A. Accurate and Efficient Model Energies for Exploring Intermolecular Interactions in Molecular Crystals. J. Phys. Chem. Lett. 2014, 5, 4249–4255. DOI: 10.1021/jz502271c
7. Turner, M. J.; Thomas, S. P.; Shi, M. W.; Jayatilaka, D.; Spackman, M. A. Energy Frameworks: Insights into Interaction Anisotropy and the Mechanical Properties of Molecular Crystals. Chem. Commun. 2015, 51, 3735–3738. DOI: 10.1039/C4CC09074H
8. Mackenzie, C. F.; Spackman, P. R.; Jayatilaka, D.; Spackman, M. A. CrystalExplorer Model Energies and Energy Frameworks: Extension to Metal Coordination Compounds, Organic Salts, Solvates and Open-Shell Systems. IUCrJ 2017, 4, 575–587. DOI: 10.1107/S205225251700848X
9. Topkaya, C.; Hökelek, T.; Aslan, S.; Özen, A. A.; Kıncal, S.; Göktürk, T.; Güp, R. Crystal Structure, Hirshfeld Surface Analysis, Crystal Voids, Interaction Energy Calculations, Energy Frameworks and Supercapacitor Applications of Isatin Hydrazone Ligand. J. Mol. Struct. 2024, 1316, 139014. DOI: 10.1016/j.molstruc.2024.139014
